# Tinnitus after treatment of vestibular schwannoma: a systematic review and comparative analysis of microsurgery and stereotactic radiosurgery

**DOI:** 10.1007/s11060-024-04935-5

**Published:** 2025-02-19

**Authors:** Ramkumar Govindaraj, Courtney Chambers, Marcus Kha, Thomas Sullivan, Sharad Chawla, Andrew Zacest, Peter Gorayski, Daniel Roos

**Affiliations:** 1 Department of Radiation Oncology, Royal Adelaide Hospital, Central Adelaide Local Health Network, Adelaide, South Australia Australia; 2 Faculty of Health and Medical Sciences, The University of Adelaide, Adelaide, South Australia Australia; 3 Women and Kids Theme, South Australian Health & Medical Research Institute, Adelaide, South Australia Australia; 4 School of Public Health, The University of Adelaide, Adelaide, South Australia Australia; 5 Department of Otolaryngology, Northern Adelaide Local Health Network, Adelaide, South Australia Australia; 6 Department of Neurosurgery, Royal Adelaide Hospital, Central Adelaide Local Health Network, Adelaide, South Australia Australia; 7 South Australia Health and Medical Research Institute, Adelaide, South Australia Australia; 8 Australian Bragg Centre for Proton Therapy and Research, Adelaide, South Australia Australia; 9 Allied Health and Human Performance Academic Unit, University of South Australia, Adelaide, South Australia Australia

**Keywords:** Vestibular schwannoma, Stereotactic radiosurgery, Microsurgery, Tinnitus, Systematic review

## Abstract

**Purpose:**

The purpose of this systematic review and meta-analysis was to compare tinnitus outcomes following microsurgery and stereotactic radiosurgery for vestibular schwannoma.

**Methods:**

The databases MEDLINE (via Ovid), EMBASE (via Ovid), Cochrane Central Register of Controlled Trials (via Ovid), SCOPUS, CINAHL (EBSCO), and Web of Science were searched for studies comparing microsurgery and radiosurgery treatment, and reporting tinnitus outcomes. Longitudinal tinnitus assessment with pre-treatment evaluation was required for inclusion. Fractionated radiotherapy treatment was excluded. Newcastle-Ottawa scale was used to assess the quality of the included studies. A separate random-effects meta-analysis was performed for the continuous, binary and ordinal tinnitus outcomes, with pooled effects described as a standardised mean difference or a log odds ratio as appropriate.

**Results:**

Thirteen studies involving 5814 patients were included in the review; 4 were prospective studies, and the rest were retrospective cohort studies. The median follow-up duration in the microsurgery and radiosurgery groups was 39.5 months and 41.1 months, respectively. Studies were diverse with respect to inclusion criteria and method of tinnitus outcome assessment. Only 4 studies reported tinnitus scores using tinnitus questionnaires, while others used Likert scale, visual analogue scale, binary (present or absent) scale or ordinal (improved, same or worse) scale. Four studies reported better tinnitus outcomes after microsurgery than radiosurgery. However, the overall quality of the studies was low, and most did not control for important confounders, such as age, tumour characteristics, and hearing impairment. Meta-analysis of continuous and binary tinnitus outcomes showed no difference between the interventions (standardised mean difference = -0.04, 95% CI -0.37 to 0.28, *p* = 0.80; log odds ratio = 0.32, 95% CI -1.11 to 1.74, *p* = 0.66). Meta-analysis of tinnitus outcomes on an ordinal scale showed microsurgery increased the odds of reporting improved tinnitus compared to radiosurgery (log odds ratio = 0.83, 95% CI 0.01 to 1.64, *p* = 0.045). Heterogeneity between the studies was high for all outcome measures (I^2^ > 56%).

**Conclusion:**

Meta-analyses of tinnitus outcomes were largely inconclusive, except when tinnitus was reported as an ordinal outcome, which favoured microsurgery. However, due to the low quality of studies and high heterogeneity, no definitive conclusions could be drawn favouring either treatment.

**Supplementary Information:**

The online version contains supplementary material available at 10.1007/s11060-024-04935-5.

## Introduction

Vestibular schwannoma is the most common benign tumour at the cerebellopontine angle. Patients with these slow-growing benign tumours present most commonly due to unilateral hearing loss, while tinnitus is less common. However, in individuals with hearing loss, the prevalence of tinnitus can be high. The pre-treatment prevalence of tinnitus in patients with vestibular schwannoma is estimated to be around 61–76% [1–6]. The 2014 Acoustic Neuroma Association survey reported that, at diagnosis, it was the second most common symptom after hearing loss, reported by 69.5% of respondents [1].

The treatment approaches for vestibular schwannoma include conservative management, radiosurgery and microsurgery. Microsurgery is generally the preferred treatment for tumours larger than 3 cm or with brainstem compression. For tumours that are 3 cm or less, there is parity in tumour control between microsurgery and radiosurgery, and the treatment decisions are increasingly based on functional outcomes with increasing use of radiosurgery [1]. However, the literature on comparative functional outcomes between microsurgery and radiosurgery is relatively sparse on tinnitus and instead heavily skewed toward hearing preservation and facial and trigeminal nerve function. Nonetheless, the impact of tinnitus on quality of life can be substantial [7–9]. Moreover, in a study evaluating patient-preferred outcomes, a significant proportion of patients (34.6%) rated reducing tinnitus as their preferred outcome, while 61% rated hearing preservation as their preferred outcome [10].

Therefore, knowledge of the longitudinal changes in tinnitus symptoms after treatment will be useful in guiding treatment, particularly in patients who are handicapped due to tinnitus. It is especially relevant due to the increasing use of conservative management in individuals with small tumours [1]; tinnitus has been observed to worsen in conservatively managed patients [11]. Knowledge of tinnitus outcomes following treatment could, therefore, underpin the decision to continue conservative management in this patient population. However, current literature on the impact of microsurgery and radiosurgery on tinnitus is mixed, and it is unclear if comparative outcomes favour any treatment.

In patients undergoing microsurgery, the impact of surgical approaches on tinnitus outcomes following microsurgery also remains a topic of debate. Among the commonly used surgical approaches, the retrosigmoid (RTS) and middle fossa (MF) aim to preserve the vestibulocochlear nerve function, while the translabrynthine (TL) approach does not, resulting in different hearing outcomes. However, the effect of these approaches on tinnitus outcomes has been inconsistent. Some studies report better outcomes after the TL approach, while others favour RTS [12, 13]. Additionally, whether these differences in tinnitus outcomes are related to the deafferentation of the vestibulocochlear nerve during surgery is still contested, as studies investigating the impact of cutting the vestibulocochlear nerve have reached opposing conclusions [14–17].

In this systematic review, we analysed studies that reported on the comparative effect of microsurgery and radiosurgery on tinnitus and present meta-analyses of the tinnitus outcomes.

## Methods

The protocol for this systematic review was registered in PROSPERO (CRD42024530708) in March 2024. Meta-analysis of Observational Studies in Epidemiology (MOOSE) guidelines were followed to report the systematic review and meta-analysis [18, 19].

The literature search included the following databases: MEDLINE (via Ovid), EMBASE (via Ovid), Cochrane Central Register of Controlled Trials (via Ovid), SCOPUS, CINAHL (EBSCO), and Web of Science. The keywords words “vestibular schwannoma”, “acoustic neuroma”, “microsurgery”, “stereotactic radiosurgery”, and “tinnitus” were used to develop a search strategy adapted to each database searched. The search strategy was peer-reviewed using the Peer Review of Electronic Search Strategies (PRESS) 2015 Evidence-Based Checklist [20] by the institution librarian before the literature search (Supplementary Material 1). The literature search was supplemented by citation tracking of included articles using SCOPUS and Google Scholar. No year limit was set; only articles in English were included. The search results were imported into Endnote and screened independently by two reviewers. A title and abstract search was first performed to identify articles retrieved for full-text inspection. Two independent reviewers scrutinised the retrieved articles for inclusion, and inconsistencies were resolved by consensus.

### Inclusion and exclusion

Studies were eligible for inclusion if the participants were adult patients with sporadic vestibular schwannoma undergoing microsurgery or radiosurgery. The microsurgery interventions eligible were retrosigmoid, translabyrinthine and middle fossa approaches; the comparator, radiosurgery intervention, included only single-fraction radiosurgery treatment using Gamma Knife or linac radiosurgery equipment.

Randomised controlled trials and non-randomised comparative effectiveness studies—prospective and retrospective cohort studies, case-control design, or cross-sectional observational studies—were eligible for inclusion. Studies were excluded if they included participants with recurrent tumours, neurofibromatosis (> 5% of participants), or fractionated radiotherapy treatment (with one minor exception – see below). Longitudinal assessments of tinnitus with pre- and post-treatment assessments were required to be reported for studies to be eligible for inclusion.

### Outcome measures

The tinnitus measures included were validated tinnitus scales – such as the Tinnitus Functional Index (TFI), Tinnitus Handicap Inventory (THI) or Tinnitus survey (TS) – visual analogue scale (VAS), Likert scale, binary outcomes such as “present or absent” or ordinal outcomes such as “improved or worse or no change”.

### Data extraction

Two independent reviewers performed data extraction. The items extracted were demographic details of the participants, tumour-related parameters, intervention details, tinnitus measures pre- and post-treatment, and study results. The data extraction was guided by a data collection form and entered into an Excel datasheet before being transferred to statistical software.

### Quality assessment

The quality assessment of the studies was performed using the Newcastle-Ottawa scale (NOS) [21]. The quality assessment tool used deviated from the one stated in the original review protocol, which was Joanna Briggs Institute Critical Appraisal Tools (JBI). The deviation occurred since the included studies were all cohort studies that could be assessed using the NOS for cohort studies, which is a more widely used tool. The JBI suite of tools would have been more suited if different types of studies had been included as initially anticipated. We opted for the NOS instead of the Risk Of Bias In Nonrandomized Studies-of Interventions (ROBINS-I) because both methods demonstrate similar reliability [22]. However, it is easier to adapt the NOS to fit the specific characteristics of observational studies being assessed. In contrast, ROBINS-I is more time-consuming and requires significantly more expertise to apply effectively [22].

The NOS assesses observational studies across three categories (selection, comparability and outcome) and awards a maximum of nine stars (Supplementary Material 2). For comparability, one star was given to studies if they controlled for the effect of age and tumour size on the tinnitus outcome at baseline or in the analysis, and two stars if the pretreatment hearing impairment was also controlled. Although the evidence for the effect of age, tumour size and hearing impairment on tinnitus outcome is not robust, it was deemed important to control these potential confounding variables. In evaluating outcomes, the NOS awards one star for independent blinded assessment, which was not suitable for patient-reported tinnitus outcomes. Therefore, studies were given a star if they used patient-reported tinnitus scale, VAS or Likert scale for outcome assessment. Studies were also given a star if they reported binary outcomes or ordinal scales, which were patient-reported. However, no stars were given if the tinnitus outcomes were clinician-reported or if it was unclear whether they were patient-reported. One star was given to studies if the follow-up was at least 24 months.

### Analysis

Continuous, ordinal and binary outcomes were pooled separately using random effects meta-analysis models. The standardised mean difference (SMD, Hedges’s g statistic) was used as the effect measure for continuous outcomes since studies used different scales (THI, Tinnitus survey and VAS), while the log odds ratio (OR) was used as the effect measure for binary outcomes. Ordinal outcomes (improved, same or worse) were converted to binary outcomes (improved versus not improved and worse versus not worse) due to evidence of non-proportional odds ratios. The pooled effect sizes were reported with 95% confidence intervals. I^2^ statistics were used to express the fraction of effect size variance due to true heterogeneity between included studies. Meta-analyses were performed using STATA 16 software.

## Results

**Fig. 1:**
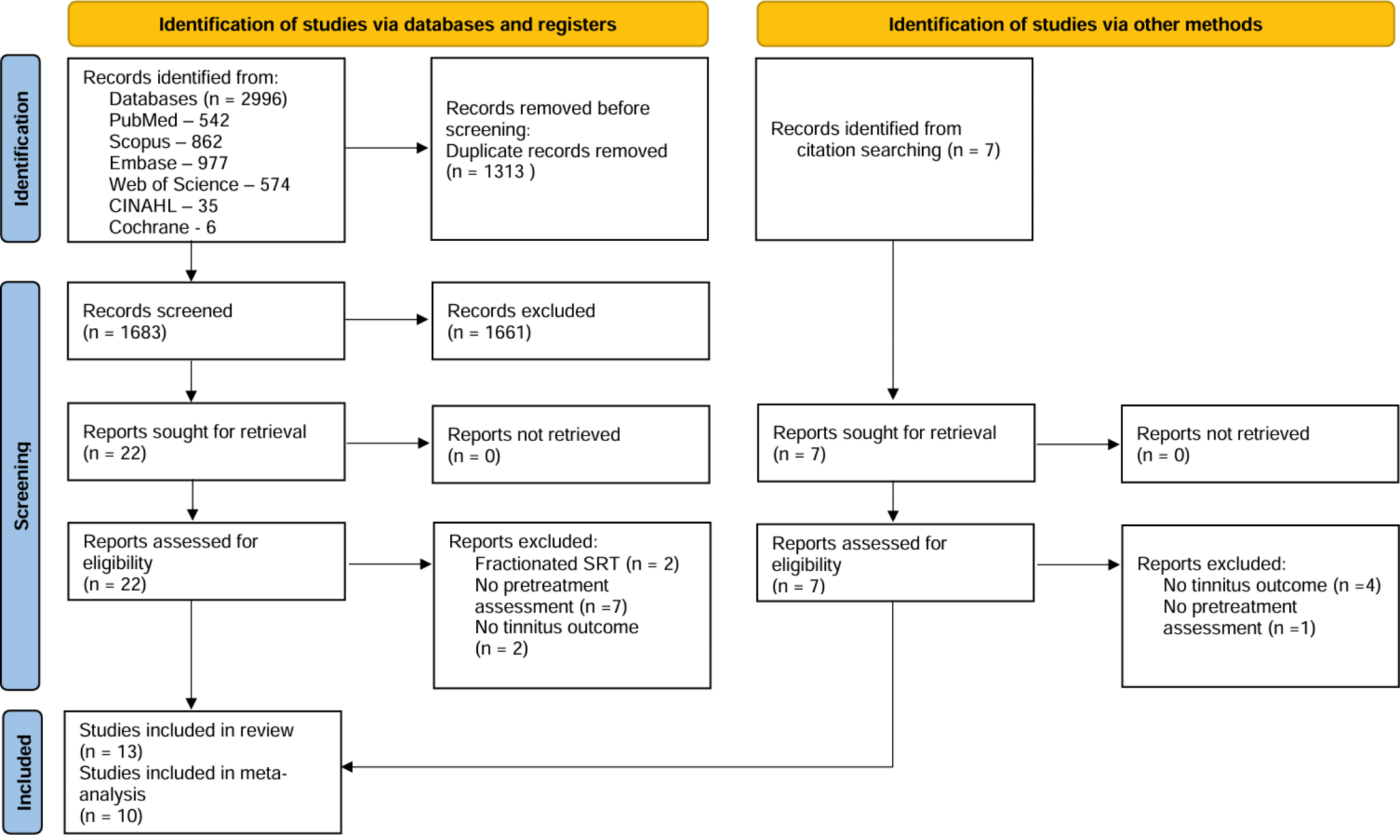
PRISMA flow diagram

The literature search of the databases yielded 1683 records after removing duplicates. Title and abstract screening of the records identified 22 relevant articles that were retrieved for full-text inspection. Citation searching identified a further 7 articles. Of the 29 articles that were examined in full, 13 fulfilled the inclusion and exclusion criteria [8, 23–34]. The PRISMA flow diagram is presented in Fig. 1.

### Characteristics of the included studies

Among the studies included, 4 were prospective cohort studies [23, 27, 31, 32] and the remaining were retrospective (Table 1). There were no randomised controlled studies. In 3 studies [8, 23, 24], the comparison was between microsurgery, radiosurgery and observation; in others, it was between microsurgery and radiosurgery. The total number of patients in the microsurgery group was 3071, and in the radiosurgery group, it was 2743.

Studies varied with respect to patients and the tumour characteristics of the population they examined. Six studies [8, 24, 25, 27, 31, 32] restricted inclusion based on tumour size, defined as either < 3, 2.5–1.5 cm or based on Koos stage. In 7 studies [23, 24, 26, 29, 32–34], the tumours were significantly larger in patients who underwent microsurgery compared to radiosurgery. In 4 of those studies [23, 26, 33, 34], this baseline difference was statistically significant (Table 1). Additionally, in all studies except for two [27, 30], the mean age of the population undergoing microsurgery was significantly lower than those undergoing radiosurgery (Table 1).

**Table 1:** Characteristics of included studies

Author	Study type	Inclusion	Comparison	Follow-up (mean in months)	Mean age (years)	Tumour size	MS (*N*)	RS (*N*)	RS details: technique and margin dose	MS approaches	Tinnitus assessment
Karpinos et al., 2002 [26]	R	Sporadic VS	MS vs. RS	MS: 31 RS: 46.7	MS: 44.8 RS: 61.6	MS: 34.8% (< 2 cm), 17.4% (> 4 cm) RS: 75% (< 2 cm), 2.7% (> 4 cm)	17	49	GK, 14.5 Gy mean	TL (52%), RTS (30%), MF (4%)	Ordinal scale (No change, better, worse)
Regis et al., 2002 [32]	P	Sporadic VS Koos stage II and III	MS vs. RS	Minimum 3 yrs (RS groups)	MS: 52 RS: 61	MS: 44.6% (Koos II), 55.4% (Koos III) RS: 66% (Koos II) 34% (Koos III)	110	97	GK, 12–14 Gy	TL (85%) and MF (15%)	Binary
Pollock et al., 2006 [31]	P	Sporadic VS < 3 cm	MS vs. RS	42	MS: 48.2 RS: 53.9	MS: 14.1 RS: 12.3	36	46	GK 12.2 Gy mean	TL (25%), RTS (69%), MF (6%)	Tinnitus Survey
Coelho et al., 2008 [25]	R	Sporadic VS with NSH and < 1.5 cm	MS vs. RS	MS: 45.5 RS: 40.2	MS: 52.8 RS: 71.25	MS: 13.7 RS: 12.3	10	12	GK	TL	Binary (present/absent) and ordinal scale
Myrseth et al., 2009 [27]	P	Sporadic VS </= 2.5 cm.	MS vs. RS	24	MS: 52.5 RS: 57.5	MS: 18 RS: 16	28	60	GK, 12 Gy mean	RTS in all expect 1 (TL)	Binary and VAS
Park et al., 2011 [29]	R	Sporadic VS with SH	MS vs. RS	MS: 49.4 RS: 43.8	MS: 49.9 RS: 59.7	MS: 35.63 RS: 19.3	15	31	GK, 14.2 Gy mean	RTS	VAS
Park et al., 2014 [30]	R	Sporadic VS with tinnitus	MS vs. RS	NA	MS: 44.5 RS: 47.8	MS: 5.05 cm^3^ RS: 4.37 cm^3^	27	19	GK, 12–13 Gy	TL	THI and VAS (ordinal)
Deberge et al., 2018 [8]	R	Sporadic VS Koos stage 1 or 2	MS vs. RS vs. OB	MS: 81 RS:57	MS: 51.4 RS: 62	MS: 13.1 RS: 14.5	43	46	GK, 12 Gy (7 treated with CK^9^)	TL (66%) and MF (34%)	THI and binary
Nuno et al., 2019 [28]	R	VS with 6 months of follow-up	MS vs. RS	MS: 39.5 RS: 38.8	MS: 50.1 RS: 56.3	NA	2029	1326	NA	NA	Binary and ordinal scale
Rizk et al., 2019 [33]	R	VS with a minimum follow-up of 6 months after MS and 1 year after RS	MS vs. RS	MS: 15.3 RS: 45.6	NA	MS: 38.1% (T3a/b) 28.6% (T4a/b) RS: 38.4 (T3a/b),15.9 (T4a/b) (Hannover classification)	275	427	GK, 13 Gy mean	RTS	Binary
Barnes et al., 2021 [23]	P	Sporadic VS	MS vs. RS vs. OB	25.2	MS: 52 RS: 61	MS: 13% > 3 cm RS: all < 3 cm	118	48	12.5 Gy median	RTS (60), TL (38%), MF (1%), transotic (1%)	Likert scale (1–10), continuous and ordinal scale
Tatagiba et al., 2023 [34]	R	Sporadic VS	MS vs. RS	MS: 72.1 RS: 82.2	MS: 47.45 RS: 59	MS: 68% (Koos III & IV) RS: 47% (Koos III & IV)	342	559	GK, 12–13 Gy	RTS	Ordinal (Grade 1–3)
Campbell et al., 2024 [24]	R	Sporadic VS < 3 cm with at least 1 year of follow-up	MS vs. RS vs. OB	MS: 30.9 RS: 33.6	MS: 56.6 RS: 64.1	MS: 17.5 RS: 9.8	21	23	NA	NA	THI

Abbreviations: MS = microsurgery; RS = radiosurgery; P = prospective; R = retrospective; VS = vestibular schwannoma; GK = Gamma Knife; CK = CyberKnife; TL = translabyrinthine; RTS = retrosigmoid; MF = middle fossa; SH = serviceable hearing; NSH = non-serviceable hearing; NA = not available; THI = Tinnitus Handicap Inventory; VAS = visual analogue scale. Tumour size in mm, unless otherwise specified

Most studies did not account for the impact of hearing impairment on tinnitus in the selection criteria, except for two [25, 29]: one study included only individuals with non-serviceable hearing, and the other included only individuals with serviceable hearing. All studies included individuals regardless of their baseline tinnitus except for one, which included only individuals with tinnitus [30].

The median follow-up for the radiosurgery group in the studies was 41.1 months, and for the microsurgery group, it was 39.5. months. In two studies, the minimum follow-up was 6 months [28, 33] and in one, it was 1 year [24]. One study did not provide details on the follow-up duration [30].

Radiosurgery treatment was delivered using Gamma Knife in all studies except 2 studies, which did not provide details (Table 1). Deberge et al. [8] included 7 patients who received the treatment using CyberKnife in three fractions. We included this study despite the mixed population because the CyberKnife proportion was small (7 out of 46), and the outcomes were not reported separately. The microsurgery technique employed in the studies was exclusively RTS in 3 studies [29, 33, 34] and TL in 2 [25, 30], the remaining studies used a mixture of TL, RTS or MF approaches. None of the studies included patients who had combination treatment (debulking surgery followed by adjuvant radiotherapy). Additionally, seven studies excluded patients who underwent both microsurgery and radiosurgery [23–25, 28, 32–34]. In one of the largest studies, 37 patients in the radiosurgery group required retreatment or surgery for recurrence [34]. The rates of salvage surgery for recurrence in the other studies were as follows: Myrseth et al. [27] reported 1.6%, Regis et al. [32] reported 3%, Pollock et al. [31] reported 4%, and Deberge et al. [8] reported 8.7%.

Tinnitus outcome was assessed and reported variedly in the studies (Table 1). Only 4 studies [8, 24, 30, 31] assessed tinnitus scores using tinnitus questionnaires: one used the Tinnitus Survey, and three used the Tinnitus Handicap Inventory. Three studies used VAS [27, 29, 30] and one Likert scale [23]. The rest of the studies assessed and reported tinnitus as a binary outcome (present or absent) or an ordinal scale (improved, same or worse). Six studies [8, 23, 25, 27, 28, 30] reported tinnitus outcomes using multiple assessment methods.

### Quality assessment

The median NOS score was 6 (range 4–8). Only 4 studies [23, 24, 29, 30] scored 8 or above, generally considered good quality (Supplementary Material 2). Three studies [23, 24, 30] controlled for the effect of age, tumour size and hearing on tinnitus outcome in the analysis; the remaining studies controlled for only one variable or none.

### Tinnitus outcome

Four studies [26, 30, 33, 34] reported results favouring the microsurgery group and none favouring the radiosurgery group. Ten studies were included in the meta-analyses; however, data could not be extracted from three studies [29, 31, 32] for inclusion in the synthesis. Specifically, Park et al. [29] and Pollock et al. [31] did not provide standard deviation values for the mean scores, and Regis et al. [32] reported the prevalence of tinnitus as percentages without providing absolute numbers. The tinnitus outcome comparisons, as reported in individual studies—including the three studies not included in the synthesis—are presented in Table 2 (Supplementary Material 3).

**Fig. 2:**
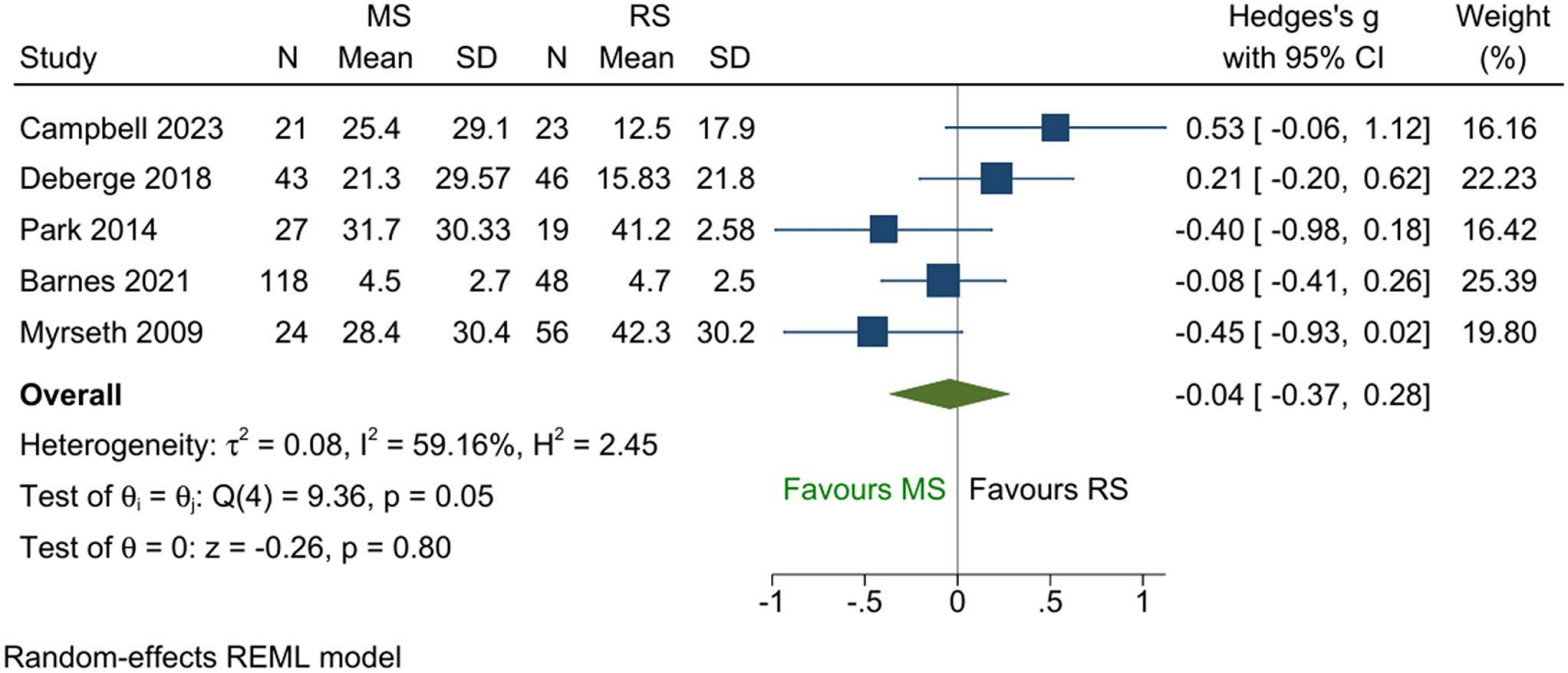
Forest plot of continuous tinnitus outcome using standardised mean difference as effect measure. A higher mean denotes a worse tinnitus outcome. Abbreviations: N = population size; SD = standard deviation; MS = microsurgery; RS = radiosurgery; REML = restricted maximum likelihood

**Fig. 3:**
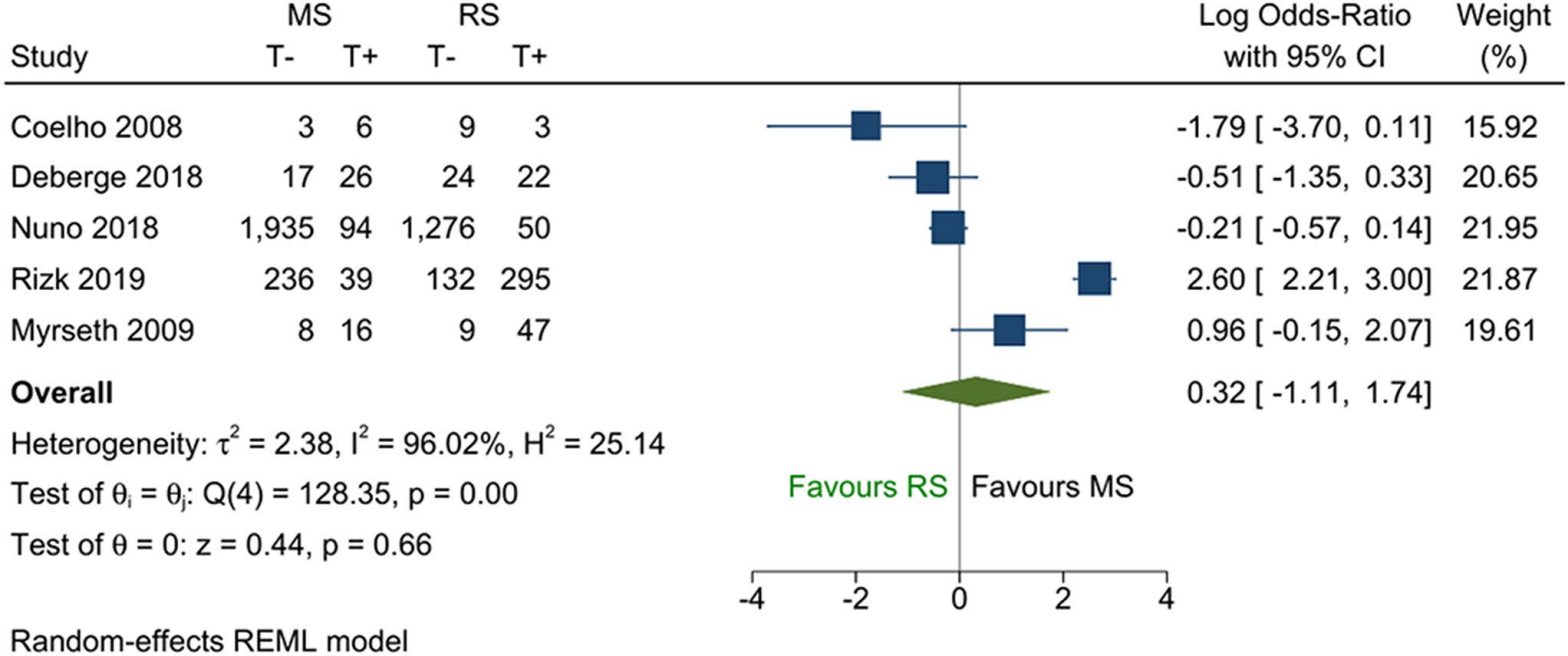
Forest plot of binary tinnitus outcome. Abbreviations: T- = tinnitus absent; T + = tinnitus present; MS = microsurgery; RS = radiosurgery; REML = restricted maximum likelihood

**Fig. 4:**
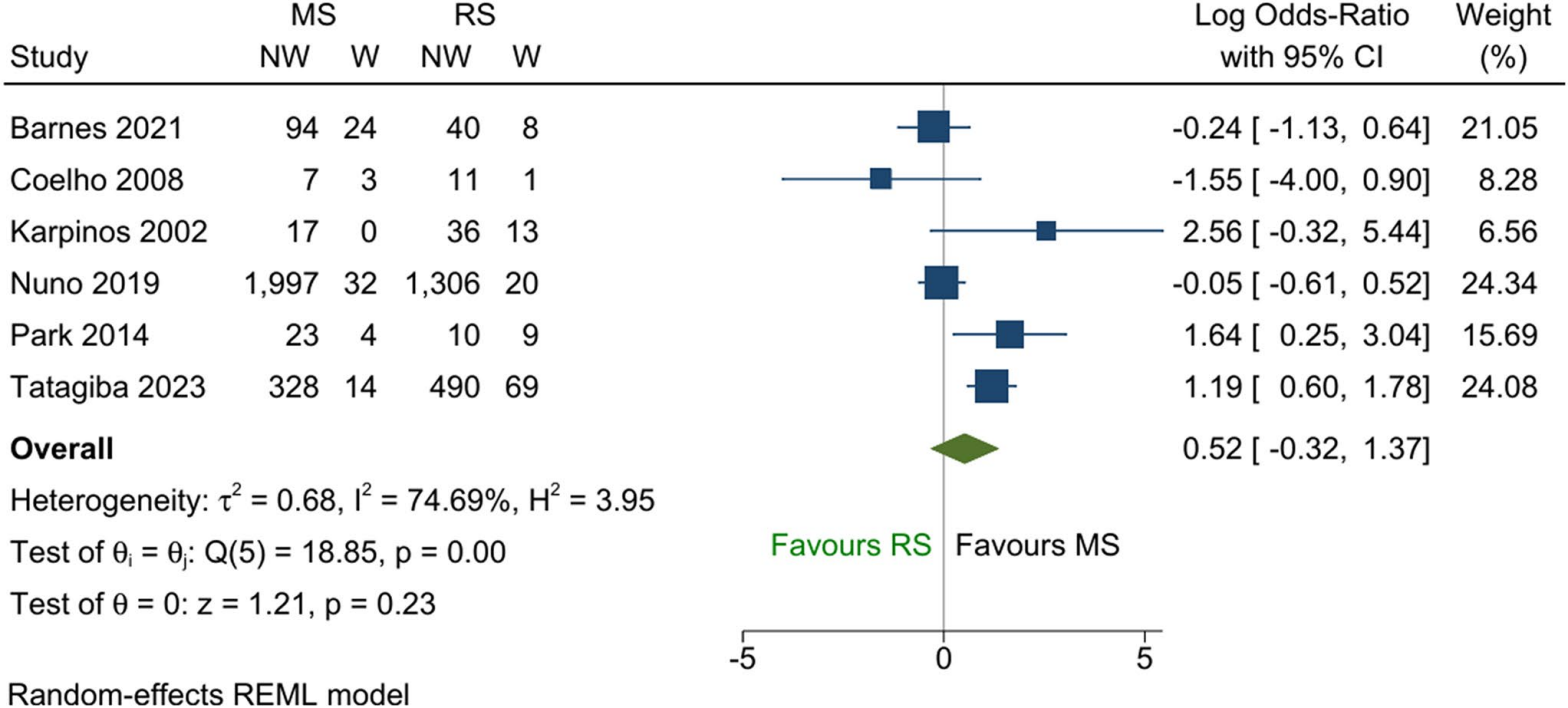
Forest plot of ordinal tinnitus outcome converted to the binary data–worse versus not worse. Abbreviations: NW = tinnitus not worse; W = tinnitus worse; MS = microsurgery; RS = radiosurgery; REML = restricted maximum likelihood

**Fig. 5:**
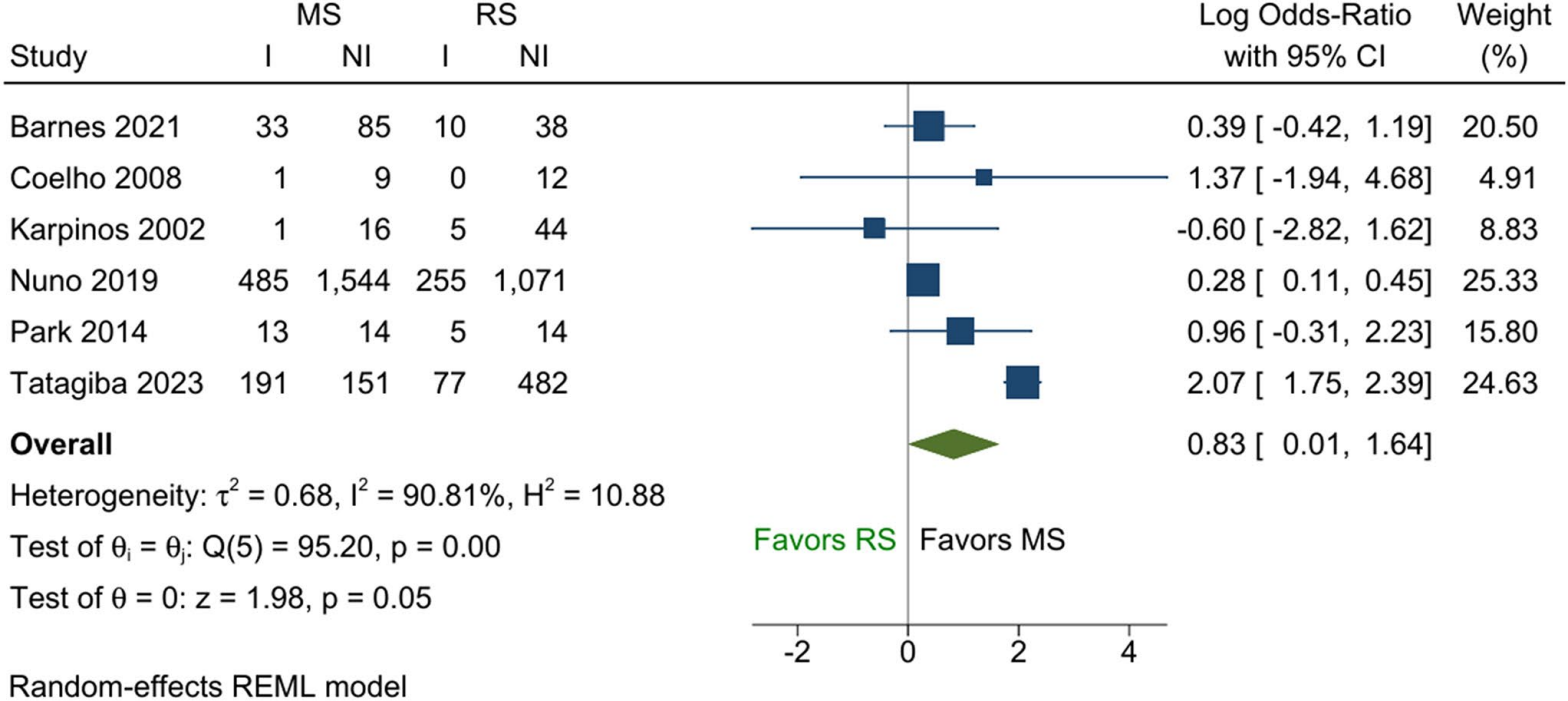
Forest plot of ordinal tinnitus outcome converted to the binary data–improved versus not improved. Abbreviations: I = tinnitus improved; NI = tinnitus not improved; MS = microsurgery; RS = radiosurgery; REML = restricted maximum likelihood

Among the five studies that reported tinnitus as a continuous outcome, no difference between the two groups was demonstrated in the pooled analysis (Fig. 2; SMD = -0.04; 95% CI -0.37 to 0.28; *p* = 0.80). Similar results were obtained from the five studies that reported binary tinnitus outcomes (Fig. 3; pooled log OR = 0.32; 95% CI -1.11 to 1.74; *p* = 0.66). The two binary comparisons generated from the ordinal outcomes are shown in Figs. 4 and 5. No difference was found between the two treatments for the comparison “not worse versus worse” (log OR = 0.52; 95% CI -0.32 to 1.37; *p* = 0.23). However, for the comparison “improved versus not improved”, the odds of improvement was 2.3 times higher for microsurgery than radiosurgery (log OR = 0.83; 95% CI 0.01 to 1.64; *p* = 0.045). There was a large variation in the effect sizes between studies due to heterogeneity, with I^2^ ranging from 59 to 96%. The lowest heterogeneity was noted for the standardised mean difference measure but was still substantial (59%).

We re-analysed continuous data after excluding the study by Deberge et al. [8] (Fig. 1) because a proportion of the population in this study received fractionated radiosurgery using CyberKnife, and the results did not change significantly (results not shown). In the meta-analysis of binary outcomes (tinnitus improved versus not improved), we evaluated the impact of excluding the study by Tatagiba et al. [34], which was heavily weighted and had a confidence interval that did not overlap with the pooled effect. Excluding Tatagiba et al. [34] reduced the heterogeneity (I^2^ < 50%), but the statistical significance remained unchanged (log odds-ratio = 0.29 95% CI 0.13 to 0.46, *p* = 0.00). It is important to note that in this meta-analysis, the two studies contributing significantly to the pooled effect size—those by Nuno [28] and Tatagiba et al. [34]—both received a score of 5 on the NOS, which is considered indicative of poor quality.

### Relation between tinnitus and hearing outcomes

All studies, except for Park et al. [30], reported on hearing outcomes (Table 2, Supplementary Material 3). The study by Coelho et al. [25] did not address hearing outcomes, as it included only patients with non-serviceable hearing loss. Most studies presented hearing outcomes in aggregate, lacking information on the relationship between hearing outcomes and tinnitus. Only Campbell et al. [24] and Park et al. [30] examined the correlation between tinnitus and preoperative hearing loss, finding no significant correlation. Consequently, it was not possible to assess the relationship between hearing outcomes and tinnitus in the studies included in the analysis.

## Discussion

### Principle findings

This systematic review presents meta-analyses of the tinnitus outcome in 13 cohort studies comparing microsurgery and radiosurgery for vestibular schwannoma. Four studies reported better tinnitus outcomes after microsurgery than radiosurgery; however, only the meta-analysis of tinnitus outcomes on an ordinal scale showed a statistically significant difference favouring microsurgery. Nonetheless, due to the significant heterogeneity between studies, pooled effects should be treated with caution. Additionally, the more reliable measure of tinnitus using the tinnitus scales, which showed less heterogeneity in the pooled analysis, did not reveal a difference between the treatments.

The included studies showed significant diversity due to variations in the tinnitus measures used and the assessment method. A range of inclusion criteria was used in the studies analysed. Some broadly included all individuals with sporadic VS, while others restricted inclusion based on factors such as tumour size, follow-up period and hearing status. The heterogeneity was evident in the large variation of the effect sizes between studies. The I^2^ statistics of more than 90% in two meta-analyses and 59% in the other indicate true variation of the effect sizes.

To our knowledge, this is the first systematic review to present a meta-analysis of the three types of outcome data. In comparison, Jakubeit et al. [35] included only 2 studies that reported tinnitus outcomes using the Likert scale and visual analogue scale in their meta-analysis. Both the studies included in the meta-analysis by Jakubeit et al. were also included in our systematic review. Jakubeit et al. found that the mean difference favoured microsurgery but concluded that the difference was not significant due to its small magnitude. In our meta-analysis of studies reporting tinnitus on a continuous scale, we analysed 5 studies using standardised mean difference as the effect measure and did not find a significant difference between microsurgery and radiosurgery. The systematic review by King et al. [36] included only single-arm cohort studies, with just two on radiosurgery, and presented a narrative review of these studies. Yakkala et al. [37] identified 3 single-arm studies—two studies on microsurgery and one on radiosurgery—that reported tinnitus outcomes. The study on radiosurgery was excluded as patients with neurofibromatosis were included, leaving no comparable radiosurgery studies. Other systematic reviews comparing microsurgery and radiosurgery did not report on tinnitus outcomes.

Cross-sectional studies were excluded from this review since they either did not perform baseline pretreatment assessment of tinnitus or relied on individuals’ recall of pretreatment tinnitus, which could lead to biased reporting. However, the cross-sectional studies that were excluded did not report a difference in tinnitus outcome between microsurgery and radiosurgery [6, 11, 38].

### Tinnitus measures

The reporting of tinnitus outcomes in the studies was varied and generally suboptimal. The ideal method of tinnitus outcome assessment would be to measure the mean change in tinnitus scores between pre- and post-treatment using a validated tinnitus questionnaire [39]. However, in only 4 studies, the tinnitus outcomes were measured using validated tinnitus scales. Three studies used binary tinnitus outcome measure (present or absent), which do not take into consideration the improvement or worsening of tinnitus that persists post-treatment. Tinnitus reported as ordinal scales, such as improved, no change, or worse, was measured differently in studies – using VAS, unvalidated measure or grading system. The use of unvalidated tinnitus outcome measures and binary outcomes, such as “present or absent” and “improved or worsened,” often lack the sensitivity needed to detect meaningful changes and can limit comparisons between studies. The high heterogeneity observed in studies reporting binary outcomes in our meta-analysis further emphasises the unreliability of the pooled effect size. In contrast, validated tinnitus questionnaires have demonstrated sufficient sensitivity to detect changes and differences in the severity of tinnitus, showing a good correlation between different questionnaires [40, 41]. Therefore, using these validated questionnaires will not only enhance the comparability between studies in the future but also provide a more accurate assessment of meaningful changes in patients’ tinnitus experience following treatment.

### Tinnitus and surgical approaches

The relation between the tinnitus outcome and surgical approach, as well as the resection of the cochlear nerve during surgery, is still uncertain since the post-operative tinnitus outcomes are inconsistent [11, 42]. The TL and RTS approaches, where the cochlear nerve is severed, have been linked to better tinnitus outcomes, with some studies reporting complete resolution of tinnitus up to 45–53% of cases after surgery [12, 43]. However, other studies found no link between cochlear nerve resection and post-operative tinnitus [15, 17, 42]. This is perhaps due to the central adaptation or maladaptation, leading to the suppression or perpetuation of tinnitus, with the location of the generation of tinnitus being less important [16, 44]. Nonetheless, cochlear nerve resection might impact the tinnitus outcome when considering the hearing status: those with worse preoperative hearing may experience better post-operative tinnitus outcomes after TL [13] and individuals with preserved cochlear nerve but post-operative hearing loss may have the worst tinnitus outcomes [14, 16].

In this systematic review, differentiating studies based on cochlear nerve status was impossible since most studies used a mixture of surgical approaches or did not provide details on the cochlear nerve status. Of the 4 studies that reported better tinnitus outcomes after microsurgery compared with radiosurgery, 2 exclusively used the RTS approach [33, 34], one TL approach [30], and the other study [26] used different techniques. Only Park et al. [30] explicitly stated the status of the cochlear nerve, which was cut in all individuals, and found improvement in tinnitus score in the microsurgery group compared with radiosurgery. Deberge et al. [8] compared tinnitus outcomes among patients who underwent microsurgery using the MF and TL approach and found no difference.

### Factors affecting tinnitus

Several factors are known to influence tinnitus, with age, tumour size, and hearing impairment being the most frequently cited. Older age is linked to higher tinnitus severity [2, 24] but also to better treatment outcomes, as older people are more likely to report improvement in tinnitus compared to younger people [16, 17, 42]. This is not a consistent finding since some studies did not find a correlation between age and tinnitus [3, 13]. However, since the mean age of the individuals in the microsurgery group was significantly lower than that of the radiosurgery group in 6 studies, this could have introduced bias in the comparison.

Pre- and post-treatment hearing impairment has been associated with unfavourable post-treatment tinnitus outcomes [38, 42], but it may not be a linear relation since profound hearing loss is associated with a lower incidence of tinnitus postoperatively [3, 12, 17, 45]. Therefore, studies evaluating tinnitus outcomes should control for the influence of hearing level. However, in this systematic review, only 5 studies controlled for hearing impairment in the selection of the population [25, 29] or the analysis [23, 24, 30].

Tumour size has also been associated with tinnitus outcome, albeit inconsistently [3, 11, 13, 42]. Smaller tumours were associated with unfavourable tinnitus outcomes post-operatively compared to larger tumours [12, 16, 17]. The inverse relation between tumour size and the post-operative tinnitus experience is significant when comparing radiosurgical and microsurgical treatment outcomes because larger tumours are more likely to be managed surgically, which could be another source of bias in the studies. In fact, the average tumour size among patients who underwent microsurgery was larger in 7 of the 13 studies included in this review. However, none of the studies in the systematic review that reported better tinnitus outcomes after microsurgery compared to radiosurgery controlled for tumour size. Only Park et al. [30] evaluated the relation between tumour volume and tinnitus outcome, finding no correlation between tumour volume and post-operative THI scores.

When comparing microsurgery and radiosurgery, it is important to consider pseudoprogression, a phenomenon associated with radiosurgery that can impact tinnitus outcomes. The literature reports a wide range of pseudoprogression rates, varying from 4.7 to 77% [46]. Most cases occur within the first 24 months, but a late peak around 36 months has also been noted after radiosurgery [46, 47]. In this systematic review, only one study explicitly reported on pseudoprogression, with a rate of 32% [34]. While most studies indicate that pseudoprogression does not lead to an increase in morbidity, some have observed a rise in hydrocephalus and cranial neuropathies, particularly affecting the facial and trigeminal nerves [46, 48, 49]. Pseudoprogression did not correlate with the worsening of tinnitus in those studies [46, 48]. The relationship between tinnitus outcomes and pseudoprogression is an understudied area, with no current research specifically addressing it.

### Limitations and future directions

This systematic review has the following limitations. First, the results of the meta-analyses must be interpreted with caution since a large proportion of the effect size variation is due to heterogeneity between studies. Second, the quality of the studies was generally low due to many factors, including suboptimal tinnitus reporting and the possibility of bias due to a lack of control of potential confounders. Third, the pooled analyses of tinnitus outcomes assessed using different tinnitus measures and varying methods may not provide the best estimate of true effects on tinnitus. Fourth, subgroup analysis to explore the sources of heterogeneity was not feasible due to the small number of studies. Lastly, these findings may not be applicable to the emerging treatment paradigm that involves managing large tumours with a combined approach of microsurgery followed by adjuvant radiosurgery.

A significant factor limiting the quality of studies on tinnitus outcomes is the lack of control for potential confounders, especially regarding tumour size and hearing outcomes. The use of various surgical approaches, along with insufficient information about cochlear nerve status, complicates the evaluation of these factors on tinnitus results. Future studies should focus on comparing patients with similar baseline characteristics, including tumour size and hearing status, as well as clearly reporting the surgical approaches used and the condition of the cochlear nerve. Additionally, comparing studies that utilise similar surgical methods may yield valuable insights into the relationship between different approaches and tinnitus outcomes.

## Conclusions

Meta-analyses of the tinnitus outcomes on a continuous and binary scale were inconclusive. When the tinnitus outcome was reported on an ordinal scale, the meta-analysis favoured microsurgery. However, the low quality of studies and high heterogeneity precluded drawing a definitive conclusion favouring any specific treatment. Given the numerous factors that can impact tinnitus outcomes and the uncertainty regarding their interactions, treatment for patients with serviceable hearing should prioritise tumour control and hearing preservation. Although microsurgery may result in improved tinnitus outcomes for certain patients with tinnitus, it remains unclear which specific factors are critical and which surgical technique is most appropriate. In the future, studies should report the mean differences in tinnitus scores using validated tinnitus scales and account for confounding factors.

## Electronic supplementary material

Below is the link to the electronic supplementary material.

Supplementary Material 1

Supplementary Material 2

Supplementary Material 3

## Data Availability

The data generated during the current study can be made available from the corresponding author upon reasonable request.
