# Supplementary material for: Tinnitus after treatment of vestibular schwannoma: a systematic review and comparative analysis of microsurgery and stereotactic radiosurgery
Source: J Neurooncol. 2025 Feb 19;172(2):347–59. doi: 10.1007/s11060-024-04935-5 (PMC11937193; doi:10.1007/s11060-024-04935-5)
Supplement: Supplementary file 1 — Supplementary Material 1 [file 11060_2024_4935_MOESM1_ESM.docx]

Search Strategy

Database: **Ovid MEDLINE(R) ALL**1946 to September 2024

| **#** | **Searches** |
| --- | --- |
| 1 | Neuroma, Acoustic/ |
| 2 | Cranial nerve neoplasm/ |
| 3 | schwannoma*.ti,ab,kf. |
| 4 | Neurilemmoma/ |
| 5 | Neurilemoma*.ti,ab,kf. |
| 6 | neurilemomma*.ti,ab,kf. |
| 7 | neurinoma*.ti,ab,kf. |
| 8 | Neuroma*.ti,ab,kf. |
| 9 | (acoustic adj1 (tumor* or tumour* or neurofibroma*)).ti,ab,kf. |
| 10 | ((cranial nerve or nerve sheath) adj2 (neoplasm* or tumo?r*)).ti,ab,kf. |
| 11 | ((cerebellopontine* adj1 angle*) and (neoplasm* or tumo?r*)).ti,ab,kf. |
| 12 | or/1-11 |
| 13 | Radiotherapy/ |
| 14 | Radiosurgery/ |
| 15 | (gamma* adj1 knife*).ti,ab,kf. |
| 16 | (cyber knife* or cyberknife*).ti,ab,kf. |
| 17 | Stereotact*.ti,ab,kf. |
| 18 | stereotaxic.ti,ab,kf. |
| 19 | Radiosurg*.ti,ab,kf. |
| 20 | Stereotaxic Techniques/ |
| 21 | Radiotherapy.ti,ab,kf. |
| 22 | Irradiat*.ti,ab,kf. |
| 23 | or/13-22 |
| 24 | microsurg*.ti,ab,kf. or Microsurgery/ |
| 25 | (Surgic* or surgery).ti,ab,kf. |
| 26 | Surgical Procedures, Operative/ |
| 27 | Otologic Surgical Procedures/ |
| 28 | neurosurgical procedures/ |
| 29 | or/24-28 |
| 30 | or/23,29 |
| 31 | and/12,30 |
| 32 | tinnitus.ti,ab,kf. or Tinnitus/ |
| 33 | and/31-32 |

The search strategy was peer-reviewed using the Peer Review of Electronic Search Strategies (PRESS) 2015 Evidence-Based Checklist by the institution librarian. The first author performed all database searches.
