# Supplementary material for: Tinnitus after treatment of vestibular schwannoma: a systematic review and comparative analysis of microsurgery and stereotactic radiosurgery
Source: J Neurooncol. 2025 Feb 19;172(2):347–59. doi: 10.1007/s11060-024-04935-5 (PMC11937193; doi:10.1007/s11060-024-04935-5)
Supplement: Supplementary file 2 — Supplementary Material 2 [file 11060_2024_4935_MOESM2_ESM.docx]

**NEWCASTLE - OTTAWA QUALITY ASSESSMENT SCALE**

**COHORT STUDIES**

Note: A study can be awarded a maximum of one star for each numbered item within the Selection and Outcome categories. A maximum of two stars can be given for Comparability

**Selection**

1) Representativeness of the exposed cohort

a) truly representative of the **average age, size or Koos stage** (describe) in the community ***(one star)***

b) somewhat representative of the **average age, size and Koos stage** in the community ***(one star)***

c) selected group of users eg nurses, volunteers

d) no description of the derivation of the cohort

2) Selection of the non-exposed cohort

a) drawn from the same community as the exposed cohort ***(one star)***

b) drawn from a different source

c) no description of the derivation of the non-exposed cohort

3) Ascertainment of exposure

a) secure record ***(one star)***

b) structured interview ***(one star)***

c) written self report

d) no description

4) Demonstration that outcome of interest was not present at start of study

a) yes (**pretreatment tinnitus assessment**) ***(one star)***

b) no

**Comparability**

1) Comparability of cohorts on the basis of the design or analysis

a) study controls for **age and tumour size** (select the most important factor) ***(one star)***

b) study controls for **pretreatment hearing level (PTA)** ***(one star)***

c) Cohorts are not comparable on the basis of the design or analysis controlled for confounders

**Outcome**

1) Assessment of outcome

a) independent blind assessment (**validated tinnitus scales, patient-reported**) ***(one star)***

b) record linkage (**Other patient-reported assessments**) ***(one star)***

c) self report (**observer reported**)

d) no description

2) Was follow-up long enough for outcomes to occur

a) yes (**24 months**) ***(one star)***

b) no

3) Adequacy of follow up of cohorts

a) complete follow up - all subjects accounted for ***(one star)***

b) subjects lost to follow up unlikely to introduce bias - small number lost - > 80% (select an adequate %) follow up, or description provided of those lost) ***(one star)***

c) follow up rate < 80 % (select an adequate %) and no description of those lost

d) no statement

Criteria for awarding stars in the NOS

| **Criteria** | **Star awarded** | **Star not awarded** |
| --- | --- | --- |
| **Selection** | | |
| Representativeness of the exposed cohort. | Average age, size or Koos stage. | Average age, size or Koos stage. |
| Selection of the non-exposed cohort. | Drawn from the same setting as the exposed cohort. | Drawn from a different setting as the exposed cohort. |
| Ascertainment of exposure | Secure records | - |
| Demonstration that outcome of interest was not present at the start of the study. | Pretreatment tinnitus assessed. | Pretreatment hearing assessment by recall. |
| **Comparability** | | |
|  | Age and tumour volume were controlled in the design or analysis.  Pretreatment hearing assessed and analysed in addition to the above | All the important factors were not controlled in the design or analysis.  No additional factors analysed |
| **Outcome** | | |
| Assessment of outcome | Validated tinnitus scales used to assess tinnitus.  Or  The subjective assessment reported by the patient. | Observer reported tinnitus outcome. |
| Was follow-up long enough for outcomes to occur | At least 24 months of mean or median follow-up. | Median or mean follow-up less than 24 months. |
| Adequacy of follow-up of cohorts | Complete follow-up of all subjects. | Follow-up rate <80% or no description of adequate follow-up. |

Quality analysis of the included studies using NOS

| Author Year | Selection | Comparability | Outcome | Total quality score | AHRQ* standards |
| --- | --- | --- | --- | --- | --- |
| Barnes 2021 | 4 | 2 | 2 | 8 | Good |
| Campbell 2023 | 4 | 2 | 2 | 8 | Good |
| Coelho 2008 | 4 | 0 | 2 | 6 | Poor |
| Deberge 2018 | 4 | 0 | 2 | 6 | Poor |
| Karpinos 2002 | 4 | 0 | 1 | 5 | Poor |
| Myrseth 2009 | 4 | 1 | 2 | 7 | Fair |
| Nuno 2018 | 4 | 0 | 1 | 5 | Poor |
| Park 2011 | 4 | 1 | 3 | 8 | Fair |
| Park 2014 | 4 | 2 | 2 | 8 | Good |
| Regis 2002 | 4 | 0 | 2 | 6 | Poor |
| Rizk 2019 | 4 | 0 | 0 | 4 | Poor |
| Tatagiba 2023 | 4 | 0 | 1 | 5 | Poor |
| Pollock 2006 | 4 | 0 | 3 | 7 | Poor |

*Agency for Healthcare Research and Quality^1^

Reference

1. McPheeters ML, Kripalani S, Peterson NB, Idowu RT, Jerome RN, Potter SA, Andrews JC. Closing the quality gap: revisiting the state of the science (vol. 3: quality improvement interventions to address health disparities). Evid Rep Technol Assess (Full Rep). 2012 Aug;(208.3):1-475. PMID: 24422952; PMCID: PMC4781280.
