# Supplementary material for: Tinnitus after treatment of vestibular schwannoma: a systematic review and comparative analysis of microsurgery and stereotactic radiosurgery
Source: J Neurooncol. 2025 Feb 19;172(2):347–59. doi: 10.1007/s11060-024-04935-5 (PMC11937193; doi:10.1007/s11060-024-04935-5)
Supplement: Supplementary file 3 — Supplementary Material 3 [file 11060_2024_4935_MOESM3_ESM.docx]

Table 2: Tinnitus and hearing outcomes in the included studies

| Author | Tinnitus assessment | Tinnitus outcomes | Tinnitus Comparison | Hearing outcomes | Comments |
| --- | --- | --- | --- | --- | --- |
| Karpinos et al., 2002^26^ | Ordinal scale (No change, better, worse) | MS: 94% of patients had no change post-treatment.  RS: 26.5% worsened, 10.2% improved, and 63.3% had no change. | Significantly more patients experienced worsening of tinnitus in the RS group (26.5% vs. 0%, p = 0.04).  . | Serviceable hearing preservation: 40% (MS) vs 44% (RS). | The mean age of patients in the MS group (44.8 yrs) was significantly less than the RS group (61.6 yrs). The tumour size was significantly smaller in the RS group. |
| Regis et al., 2002^32^ | Binary | New-onset tinnitus was seen in 50% of RS patients and 40% of MS patients post-treatment. 84% of RS and 67% of MS patients with tinnitus pretreatment had tinnitus post-treatment. | Reduction of tinnitus: 16% vs 33% for RS and MS, respectively – not statistically significant. | Functional hearing preservation (GR 1 or 2): 5% (MS) vs 40% (RS). | The mean age of patients in the MS group (52 years) was less than the RS group (61 years). MS group had more patients with stage III tumours (55% vs 34%) and tinnitus. |
| Pollock et al., 2006^31^ | Tinnitus Survey | Mean baseline score: 11.6 (MS) vs 9 (RS)  Mean scores at last follow-up: 11.6 (MS) vs 10 (RS) | There was no significant difference between mean scores at the last follow-up (p=0.29). | AAO-HNS Class A or B: 5% (MS) vs 63 % (RS). | The mean age of patients in the MS group (48.2 yrs) was significantly lower than in the RS group (53.9 yrs). |
| Coelho et al., 2008^25^ | Binary (present/absent) and ordinal scale | 2 of 12 and 5 of 9 patients in the RS and MS group had pretreatment tinnitus. | MS: 1 new-onset, 1 improved, 2 worsened, 2 patients had changes in tinnitus post-surgery.  RS: 9 of 10 patients without pretreatment tinnitus had no change. | Included only patients with NSHL. | The mean age of the patients in the MS group (52.8 yrs) was less than the RS group (71.25 yrs). |
| Myrseth et al., 2009^27^ | Binary and VAS | Mean change in VAS:  MS: - 6.9  RS: - 5.9  Patients with tinnitus pre- and post-treatment:  MS: 85.7 and 66.7%  RS: 81.7 and 83.1% | No significant difference in change in VAS between groups at 2 years. | Serviceable hearing preservation: 0% in (MS) vs 68% (RS). | The VAS was lower in the MS group than in the RS group at baseline and 1 year (p=0.03). |
| Park et al., 2011^29^ | VAS | Tinnitus scores pre- and post-treatment:  MS: 4.9 and 3.1  RS: 5.3 and 3.5 | Decrease in tinnitus scores: 37% (MS) vs 34%(RS)  No patient had an increase in score. | Serviceable hearing: 7% (MS) vs 45% (RS). | The mean age of patients in the MS group (49.9 yrs) was lower than the RS group (59.7 yrs). The mean tumour size was lower in the RS group (19.3 mm) than in the MS group (35.6 mm). |
| Park et al., 2014^30^ | THI and VAS (ordinal) | Mean change in THI scores:  MS: 51.25 to 31.7 (p=0.006)  RS: 15.29 to 41.2 (p<0.05) | Tinnitus scores (THI and VAS) in the MS group significantly decreased, and scores in the RS group significantly increased. | NA | Definition of significant change: at least 20% change in THI and 10 scores in VAS.  Vestibulocochlear nerve cut during MS. |
| Deberge et al., 2018^8^ | THI and binary | Patients with tinnitus pretreatment and post-treatment:  MS: 39.5% and 60.5%  RS: 39.1% and 47.8%  Mean THI scores post-treatment:  MS: 21.30  RS: 15.83 | There was no significant difference in THI scores between groups. | GR I/II: 9.3% (MS) vs 23.9 % (RS). | The mean age of patients in the MS group (51.4 yrs) was lower than the RS group (62 yrs).  No pretreatment THI scores were available.  7 patients were treated with CK. |
| Nuno et al., 2019^28^ | Binary and ordinal scale | Patients with tinnitus pre- and post-treatment:  MS: 26.9% and 4.6%  RS: 21.5% and 3.8% | Tinnitus improved in both groups. A greater improvement in the MS group: 23.9% vs 19.2%. Tinnitus morbidity at 6 months: OR 1.06 (0.71-1.52). | MS group had a higher rate of hearing loss: Odds ratio = 2.15 (95% CI:1.82-2.52) | Provider reported tinnitus assessment.  Tinnitus was assessed at 6 months post-treatment.  The mean age of patients in the MS group (50.1 yrs) was lower than the RS group (56.3 yrs). |
| Rizk et al., 2019^33^ | Binary | Patients with tinnitus pre- and post-treatment  MS: 77.9% and 14.1%  RS: 71.9% and 69.1% | The MS group had fewer patients with tinnitus post-treatment than the RS group (p=<0.001). | GR I or II: 25% (MS) vs 33.8% (RS). | Longer follow-up in the RS group (45.6 months) vs the MS group (15.3 months).  Tumour size was significantly larger in the MS group. |
| Barnes et al., 2021^23^ | Likert scale (1-10), continuous and ordinal scale | Mean change in tinnitus scores  MS: -0.1(CI -0.7 - 0.4)  RS: 0.6 (CI -0.2 - 1.5)  Tinnitus worsening: 20% (MS) vs 17%(RS)  Tinnitus improved: 28%(MS) vs 21% (RS) | Within-group mean change was not significant. | AAO-HNS Class A or B: 19% (MS) vs 27% (RS). | The mean age of patients in the MS group (52 yrs) was lower than the RS group (61 yrs). Tumour size was significantly larger in the MS group.  Definition of clinically important change: worsening or improvement of the score by >/=2. |
| Tatagiba et al., 2023^34^ | Ordinal (Grade 1-3) | Improvement in tinnitus post-treatment:  56% (MS) vs 14% (RS)  Worsening of tinnitus post-treatment:  4% (MS) vs 12% (RS) | More patients with worse tinnitus post-treatment in the RS group than in the MS group; more patients had improvement in the MS group (p= <0.001). | Hearing loss for patients with baseline GR I and II: 37% (MS) vs 54% (RS). | The mean age of the patients in the MS group (47.5 yrs) was lower than that of the RS group (59 yrs). MS group had significantly more Koos IV and functional hearing. Definition of significant change: a change of 1 point in grade. |
| Campbell et al., 2024^24^ | THI | Mean change in tinnitus score  MS: 2.5 (22.9 to 25.4)  RS: 2.5 (10 to 12.5) | Changes in scores were not statistically significant. | There was no correlation between the presence of hearing loss and tinnitus handicap inventory. Did not report other hearing outcomes | Age was a significant predictor for THI scores.  The mean age of the patients in the MS group was lower (56.6) than in the RS group (64.1), and the tumour size in the MS group was significantly larger. |

Abbreviations: MS =microsurgery; RS = radiosurgery; CK = CyberKnife; THI = Tinnitus Handicap Inventory; VAS = visual analogue scale; OR = odds ratio; CI = confidence interval; NSHL = non-serviceable hearing loss; AAO-HNS = American Academy of Otolaryngology-Head and Neck Surgery; GR = Gardner-Robertson grading scale.
